# Presence of Potentially Infectious Human Enteric Viruses and Antibiotic Resistance Genes in Mussels from the Campania Region, Italy: Implications for Consumer’s Safety

**DOI:** 10.1007/s12560-025-09635-5

**Published:** 2025-05-15

**Authors:** Iolanda Venuti, Enric Cuevas-Ferrando, Irene Falcó, Inés Girón-Guzmán, Marina Ceruso, Tiziana Pepe, Gloria Sánchez

**Affiliations:** 1 Department of Veterinary Medicine and Animal Production, University of Naples Federico II, Via F. Delpino, N. 1, 80137 Naples, Italy; 2 Institute of Agrochemistry and Food Technology, IATA-CSIC, Av. Agustín Escardino 7, 46980 Paterna, Valencia Spain; 3 Department of Microbiology and Ecology, University of Valencia, Valencia, Spain

**Keywords:** Foodborne viruses, Viability RT-qPCR, ARGs, Food safety

## Abstract

This study presents a comprehensive assessment of viral contamination and antibiotic resistance genes (ARGs) presence in mussels (*Mytilus galloprovincialis*) (*n* = 60) collected from retail stores in the Campania region (Italy). High prevalence of human noroviruses (HuNoV) genogroup I (GI) (77%) and genogroup II (GII) (40%), rotaviruses (RV) (60%), and astroviruses (HAstV) (25%) was found, with average levels of 4.34, 5.09, 5.05, and 4.00 Log genome copies (GC)/g, respectively. All samples tested negative for hepatitis A and E viruses. Viral faecal contamination indicators, including somatic coliphages (88%, 3.62 mean Log plaque forming units (PFU)/100 g) and crAssphage (50%, 3.72 mean Log GC/g), showed strong correlations (*ρ* > 0.65, p-value < 0.05) with HuNoV GII, HAstV, and RV concentrations in mussels. The study also investigated the presence of respiratory viruses, with all samples testing negative for SARS-CoV-2, respiratory syncytial virus, and influenza A virus.

Furthermore, a capsid-integrity RT-qPCR assay was applied to selected positive samples, confirming the presence of potentially infectious viruses and underscoring the associated risks to consumers.

Additionally, ARGs were detected by qPCR, targeting beta-lactams, quinolones, and chloramphenicol resistance genes in both the total and the bacteriophage fractions of selected samples.

Overall, this study emphasizes the importance of continued surveillance and strategic interventions to mitigate public health risks associated with the consumption of contaminated bivalve molluscan shellfish (BMS), which may imply the dissemination of infectious enteric viruses and ARGs within communities.

**Supplementary Information:**

The online version contains supplementary material available at 10.1007/s12560-025-09635-5.

## Introduction

Foodborne viral pathogens pose a significant threat to global public health and economy, often causing outbreaks of gastroenteritis or hepatitis and presenting substantial risks to human well-being. Among them, human noroviruses (HuNoV) genogroup I and II (GI and GII), rotaviruses (RVs), astroviruses (HAstVs), and hepatitis E (HEV) and A (HAV) viruses, predominantly originating from human faecal matter, exhibit robust transmission capabilities and remarkable environmental resistance. Furthermore, traditional wastewater treatment methods are known to face significant challenges in achieving complete virus elimination (Sano et al., 2016) and during substantial rain events or dry water overflows, such as snowmelt, tidal infiltration, or system failures and obstructions, substantial quantities of untreated wastewater can be discharged (Ahmed et al., 2020). This discharge contaminates water bodies and facilitates the spread of viral pathogens to bivalve molluscan shellfish (BMS) harvesting areas, posing a risk to individuals who consume contaminated BMS (Campos & Lees, 2014; La Rosa et al., 2017).

Waterborne viruses, originating from human faecal matter, can adhere to solid materials within the water column or accumulate in sediments, thereby increasing their transmissibility through environmental fluids (Hassard et al., 2016). Consequently, filter-feeding aquatic species, such as mussels and oysters—commonly harvested for human consumption and often eaten raw or mildly cooked—may retain these viruses and facilitate enteric illness outbreaks within communities (Landry et al., 1983; Lowther et al., 2012). In Italy, raw mussels are largely consumed in Bari and, generally, in Puglia, especially at Christmas and Easter (Prato et al., 2004; Venugopal & Gopakumar, 2017). This way of consuming mussels is also found in other parts of Italy, but to a lesser extent, increasing the risk of infection in the whole country. Considering their ability to filter large volumes of water, these organisms can retain pollutants, serving as indicators of environmental contamination and assisting in evaluating overall water quality. Therefore, BMS have been studied to act as indicators of pollution (Lees et al., 2000; Suffredini et al., 2011). The deficiencies in wastewater treatment further heighten the risk, potentially allowing not only enteric viruses but also respiratory viruses to reach the aquatic environment (Guerrero-Latorre et al., 2020; Wurtzer et al., 2020). Recent studies have detected severe acute respiratory syndrome coronavirus 2 (SARS-CoV-2), Influenza A virus (IAV), and Respiratory Syncytial Virus (RSV) in wastewater samples (Boehm et al., 2023; Toribio-Avedillo et al., 2023; Girón-Guzmán et al., 2024), raising concerns about the potential contamination of BMS (Mancusi et al., 2022; Polo et al., 2021). For instance, Mancusi et al. (2022) identified SARS-CoV-2 in 15.1% of mussels collected in the Campania region during a critical period from September 2019 to April 2021. This timeframe coincided with a surge in viral discharges of seawater, correlating strongly with the increasing number of COVID-19 cases in the region (Lombardi et al., 2023; Perrella et al., 2022). In this context, detecting both enteric and respiratory viruses (SARS-CoV-2, IAV, and RSV) becomes crucial for understanding and mitigating potential public health risks associated with contaminated water sources (Dlamini et al., 2024).

The current regulatory framework, as outlined by Commission Regulation 2073/2005, primarily relies on microbiological criteria for BMS, with a focus on bacterial indicators like *Escherichia coli*. BMS production areas are categorized as A, B, or C, with the classification determined by *E. coli* levels in BMS (Regulation (EU) 2019/627) (EC, 2005; EC, 2019). Specific post-harvest treatments, such as depuration and relaying, are mandatory for BMS harvested from production areas classified as B or C before BMS can enter the market. While this framework effectively addresses bacterial risks associated with microbiological contamination in BMS, extensive research has revealed that bacteria serve as inadequate indicators for assessing viral contamination (Hunt et al., 2023; Oh et al., 2015; Sharp et al., 2021) and commercial depuration treatments cannot guarantee the complete absence of viruses in BMS (Oliveira et al., 2011; Polo et al., 2014; Romalde et al., 2002). In essence, the current system, which classifies and intervenes based on *E. coli*, is not as effective in managing the risks associated with viruses. Thus, reliable indicators of viral faecal contamination are crucial. CrAssphage, a bacteriophage associated with the human gut microbiome, has emerged as a promising marker for faecal contamination in environmental samples, including BMS (Farkas et al., 2019; Gyawali et al., 2021; Stachler and Bibby, 2014). Integrating such novel indicators along with somatic coliphages into surveillance strategies is essential for expanding our understanding of the epidemiological spread of enteric viruses in BMS and assessing potential contamination risks. Additionally, for a realistic risk assessment, it is essential to understand the infectivity potential of the detected viruses. This includes considering various factors such as the health status of consumers, the viral infectious dose, and the likelihood of virus propagation. Capsid-integrity assays, which evaluate the structural soundness of viral capsids, offer a tool to estimate viral infectivity (Randazzo et al., 2016). These assays, more economical and less time-consuming than traditional cell culture assays, present an attractive option for routine laboratory use (Razafimahefa et al., 2021). Notably, the enhanced version of propidium monoazide (PMA), PMAxx, has recently been demonstrated to share the same spectral properties as PMA while exhibiting superior effectiveness in discriminating between infectious and non-infectious viruses through capsid-integrity (RT-)qPCR (Randazzo et al., 2016, 2018a, 2018b). This advancement in methodology holds promise as a valuable tool for the future detection of infectious viruses, enabling a more accurate assessment of viral hazards associated with BMS. However, it should be considered that this is a capsid-integrity assay, serving as an approach for assess the potential infectiousness of the viral particle, and cell culture remains the only fully reliable method for assessing viral infectivity.

Beyond viral contamination, the rise and spread of antibiotic resistance genes (ARGs) within the food supply chain pose a growing and pressing public health issue. These ARGs, which grant resistance to antibiotics, play a crucial role in the growth of antibiotic-resistant bacteria, restricting treatment choices and worsening the challenges of infectious diseases (Koutsoumanis et al., 2021). Moreover, bacteriophages have been recognized as potential carriers facilitating the dissemination of ARGs (Gunathilaka et al., 2017; Jebri et al., 2021). In this sense, conventional wastewater treatments aim to achieve a substantial decrease in bacterial concentrations (Marín et al., 2015). However, as in the case of pathogenic enteric viruses, the effectiveness of these treatments does not necessarily lead to significant reductions either in the quantity of resistant bacteria or in ARGs (Huang et al., 2012; Novo & Manaia, 2010), as their impact on resistant bacteria and ARGs varies significantly based on the specific environmental conditions, pollutant densities, and operational parameters, as reviewed in Calero-Cáceres et al. (2019).

In the context of phages carrying ARGs, these genes exhibit substantial persistence even after undergoing disinfection treatments and natural degradation due to their protection within the viral capsid (Calero-Cáceres & Muniesa, 2016). These characteristics provide them with an enhanced potential for horizontal gene transfer events among bacteria. Understanding the presence of ARGs in both, bacterial and phage fractions, is essential for comprehending potential risks and devising strategies to mitigate the spread of antibiotic resistance within the BMS production chain.

Overall, the objective of this study was to evaluate the microbiological risk associated with mussel consumption for Italian consumers, particularly given the cultural practice of consuming mussels raw in certain regions, by analysing common foodborne viruses and recently proposed viral faecal indicators. The study also aimed to assess the potential utility of a capsid-integrity protocol to approximate the infectiousness of detected viruses, providing an innovative approach to better understand viral hazards. Furthermore, this work aimed to take advantage of the laborious sample processing required for microbiological risk assessment to extract the maximum information by exploring the capability of mussels as indicators for the spread of ARGs and respiratory viruses, enhancing their value as tools for environmental and public health monitoring.

## Material and Methods

### Virus Concentration and Nucleic Acid Extraction from Mussels

**Fig. 1:**
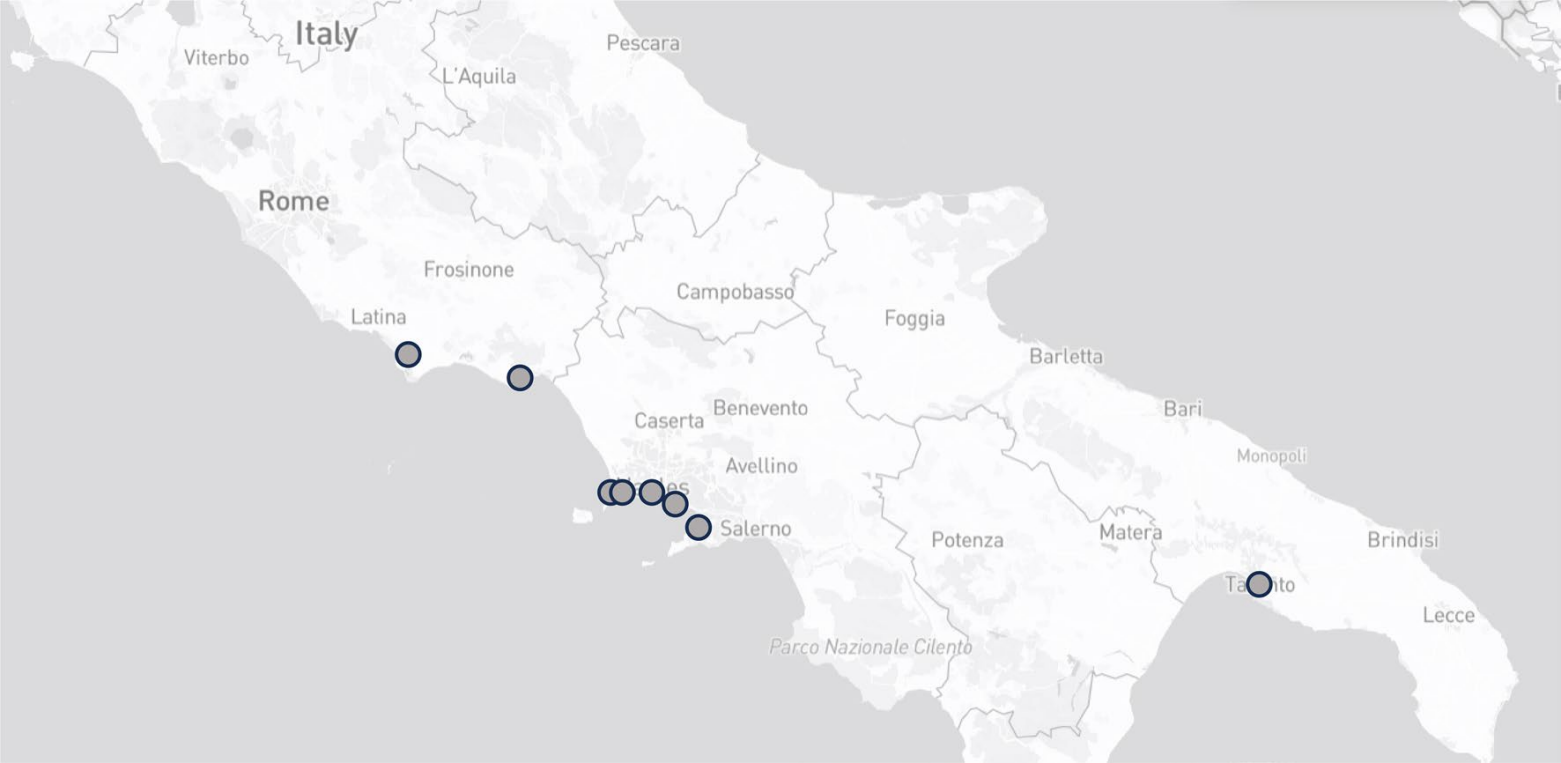
Geographical map of bivalve molluscan shellfish production areas linked to retail samples in the Campania Region, Italy

Mediterranean mussels (*Mytilus galloprovincialis*) were obtained from local retail stores in the Campania region (Southern Italy) between April and June 2023 (Fig. 1). A total of 60 batches of fresh BMS, each comprising a minimum of 25 mussels, were purchased. The production area of all the batches was within Italy, with 38 batches originating from the Campania region, 18 batches from the Lazio region, and 4 batches from the Puglia region (Fig. 1). All the collected samples were promptly frozen and shipped under controlled temperature conditions to the Institute of Agrochemistry and Food Technology (IATA-CSIC) (Spain) laboratories for subsequent analysis.

**Fig. 2:**
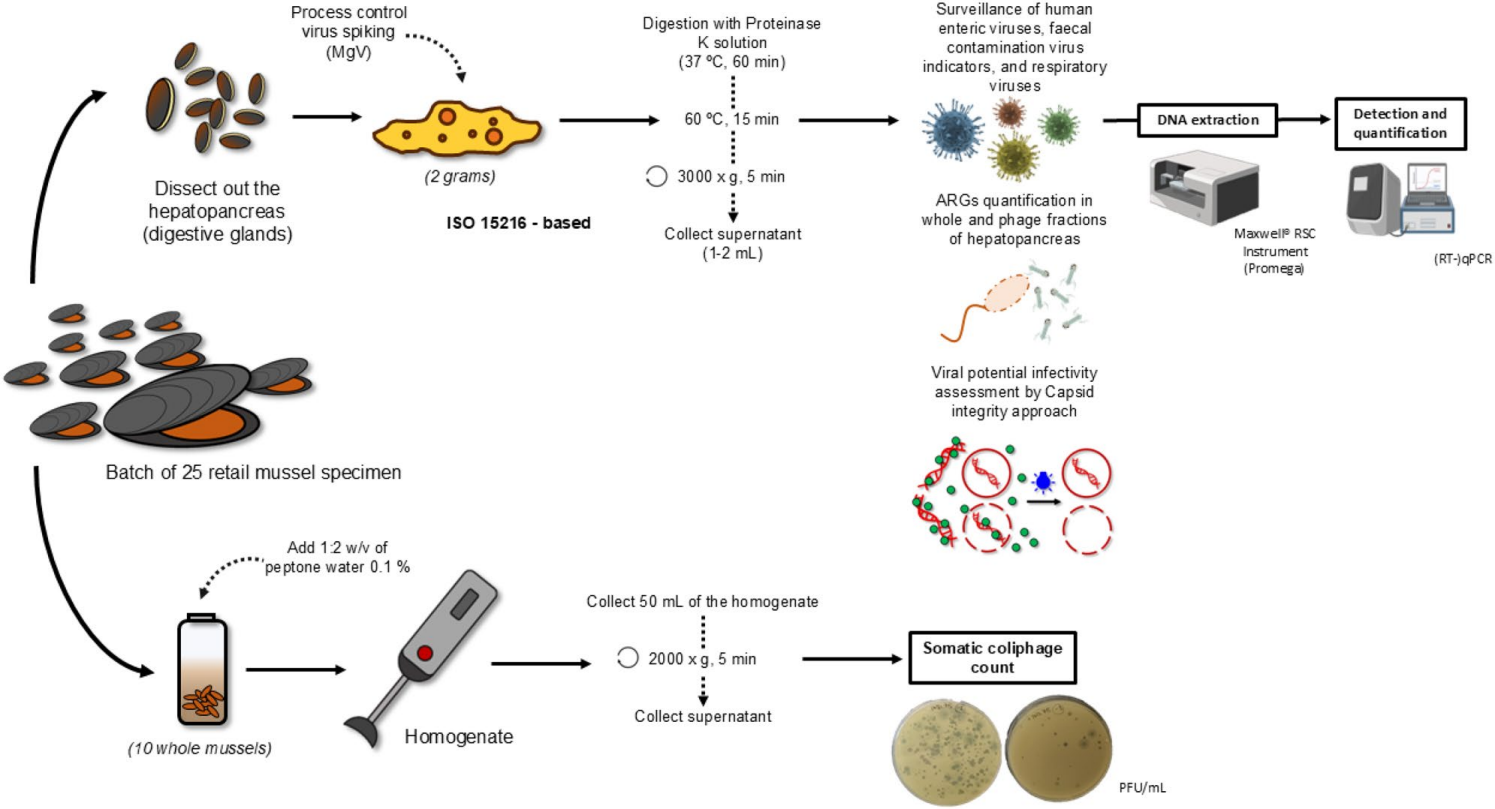
Workflow followed to analyse human enteric viruses, respiratory viruses, viral faecal indicators and antibiotic resistance genes (ARGs) in mussel samples. The somatic coliphage count assay was performed using whole mussel homogenates, whereas virus isolation and capsid-integrity assays were conducted on the digestive glands (hepatopancreas) of the mussels

Virus isolation from mussel hepatopancreas was carried out following the ISO 15216 protocol; however, the nucleic acid extraction method deviated from the procedure described in the ISO 15216. To ensure representative sampling, hepatopancreas samples were pooled from 25 mussels within each batch, from which 2 g of tissue was selected for further analysis. Mengovirus (MgV) vMC0 (CECT 100000) was used as process control. The final concentrate (1–2 mL) was either processed immediately or stored at – 80 °C (Fig. 2).

Nucleic acid extraction from mussel concentrates (300 μL) was performed using the Maxwell® RSC Instrument (Promega, Spain) with the Maxwell RSC Pure Food GMO and authentication kit (Promega), utilizing the “Maxwell RSC Viral total Nucleic Acid” running program (Pérez-Cataluña et al., 2021), resulting 100 µL of eluate.

### Detection and Quantification of Enteric and Respiratory Viruses, and Viral Indicators

Levels of HuNoV GI and GII, HAstV, RV, HAV, and HEV were determined using the RNA UltraSense One-Step kit (Invitrogen, USA) as previously described (Randazzo et al., 2018a). The occurrence of crAssphage was established using the qPCR Premix Ex Taq™ kit (Takara Bio Inc., USA) using primers and conditions described by Stachler et al. (2017). Viral detection of MgV was performed by RT-qPCR using the One-Step PrimeScript™ RT-PCR Kit (Perfect Real Time) (Takara Bio Inc., USA). Viral detection of IAV was determined using primers from CDC (Research Use Only CDC Influenza SARS-CoV-2 “Flu SC2” Multiplex Assay Primers and Probes) and as described by CDC (protocol of real-time RT-PCR for influenza A(H1N1)) by using PrimeScript RT-PCR kit (Takara Bio Inc., USA) (CDC, 2022). RSV detection was performed as previously described (Sanghavi et al., 2012). SARS-CoV-2 detection was achieved by targeting the N1 region of the nucleocapsid gene. The One-Step PrimeScript™ RT-PCR Kit was used with N1 primers and conditions described by CDC (CDC, 2019).

Different controls were used in all assays: negative process control consisting of phosphate-buffered saline (PBS); whole process control to monitor the method efficiency of each sample (MgV spiking); and positive (reference material) and negative (RNase-free water) RT-qPCR controls. A table, featuring primers, probes, PCR conditions, limit of quantification (LOQ/L), and limit of detection (LOD/L) for all targeted viruses in this work, is available in the Supplementary Material (Table S1).

Synthetic gBlock gene fragments (Integrated DNA TechnoLogies, Inc., USA) comprising the PCR-targeted regions of crAssphage, HuNoV GI and GII, HAstV, RV, HAV, and HEV were used to prepare standard curves (Table S4). For IAV and RSV quantification, Twist Synthetic InfluenzaV H1N1 RNA control (part number: 103001) and purified RNA of RSV (Vircell, S.L) were used.

The qPCR analyses were performed using a LightCycler (Roche Diagnostics, Switzerland) and a QuantStudio 5 (Thermo Fisher Scientific, USA) system.

The presence of somatic coliphages was determined as previously described (Polo et al., 2014). Briefly, a pool of 10 whole mussels was homogenized with peptone water (PW), followed by centrifugation at 2000 × g for 7 min. Subsequently, 1 mL of the supernatant was employed for the enumeration of somatic coliphages using a commercial Bluephage Easy Kit for Enumeration of Somatic Coliphages (Bluephage S.L., Spain), following manufacturer’s instructions (Fig. 2).

### Capsid-Integrity Assay in Mussel Samples

To assess the integrity of viral capsids in mussel samples, a protocol based on capsid permeability to PMAxx (Randazzo et al., 2018a) was evaluated in a subset of samples that had tested positive for more than one of the targeted pathogenic viruses (*n* = 8, S1—S8, corresponding to M21, M27, M28, M29, M38, M41, M42, and M43 samples, respectively). Briefly, 300 µL of bivalve molluscan shellfish supernatant after proteinase K digestion (Fig. 2) was placed in DNA LoBind 1.5 mL tubes (Eppendorf, Germany) and the photoactivable dye PMAxx™ (Biotium, USA) was added at 100 µM along with 0.5% Triton 100-X (Thermo Fisher Scientific, Spain). Then, two cycles of incubation in the dark at room temperature (RT) for 10 min at 150 rpm plus exposure to photoactivation for 15 min using a Led-Active Blue system (GenIUL, Spain) were performed prior to nucleic acid extraction using the Maxwell system as described above. Thermally inactivated bivalve molluscan shellfish supernatant concentrate samples (treated at 95 ºC for 10 min) were also processed in parallel as damaged-capsid positive control.

### ARG-Presence in Mussel Samples

The presence of ARGs was assessed in both the total and the phage fractions of hepatopancreas concentrates. Phages carrying ARGs were purified from these samples as previously described by Larrañaga et al. (2018) with some modifications. Briefly, 600 µl of mussel hepatopancreas concentrates diluted at a 1:2 ratio with PBS were filtered through 0.22 μm low protein binding cellulose acetate (CA) membranes (Corning Costar Corp., USA). The filtrates were treated with chloroform (10% v/v) and shaken for 5 min at RT. Then, the two-phase mixture was separated by centrifugation at 4000 × g for 10 min. The collected aqueous phase was treated with 100 U of DNAse I (Sigma-Aldrich, USA) at 37 °C for 1 h using the reaction buffer provided by the manufacturer. DNase I was then inactivated by heating at 75 °C for 5 min. Additionally, in parallel, the same hepatopancreas concentrates were diluted 1:2 in PBS to assess the presence of ARGs in the total fraction of the sample, including bacteria. Finally, DNA extraction was performed using the Maxwell RSC Pure Food GMO and authentication kit (Promega) as described above.

### ARGs Quantification Methods

The levels of the ARGs *bla*_CTX-M_, *qnrB*, and *catl* were assessed by qPCR in a subset of hepatopancreas samples that exhibited high levels of somatic coliphages (*n* = 8, corresponding to M22, M28, M39, M41, M42, M43, M47, and M48). ARGs were amplified by using the QuantStudio 5 Real-Time PCR Instrument with the KAPA SYBR FAST 2 × mastermix kit (Kapa Biosystems, SA). Primers used for ARGs amplification are described in Table S3. Results were analysed with the Applied Biosystems StepOne Instrument program and Quantstudio™ Design & Analysis software version 2.6 (desktop and Thermo Fisher™ Connect), respectively. Reaction mixtures had a final volume of 10 μL containing 2.5 μL of the extracted DNA. All samples were analysed in duplicate. In all cases, a non-template control (NTC) was included using 2.5 μl of DNAse free water instead of the DNA template. Standard curves of tenfold serial dilutions were performed in triplicate (Table S4).

### Statistics

Results were statistically analysed using GraphPad Prism Software and the significance of differences was determined on the ranks with a one-way analysis of variance (ANOVA) and Tukey’s multiple comparison tests. Spearman’s rank correlation analyses were conducted to assess the strength of the relationship between viral titers of enteric viruses and viral faecal contamination indicators. In all cases, a *p*-value < 0.05 was deemed significant.

## Results

### Prevalence of Human Enteric and Respiratory Viruses and Viral Faecal Contamination Indicators

In this study, an extensive analysis was conducted to ascertain the concentration of various human pathogenic enteric viruses in the collected samples. Mengovirus recovery values for all analysed samples ranged from 2.58% to 31.95% (Table S2). Thus, all extraction efficiencies exceeded the > 1% recovery rate required by ISO 15216. Notably, HuNov GI was identified in a significant proportion of the samples, with a prevalence of 77% (46/60) and a mean concentration of 4.34 ± 0.32 Log genome copies (GC)/g of hepatopancreas. Furthermore, HuNoV GII was detected in 40% (24/60) of the analysed samples with a mean concentration of 5.09 ± 0.37 Log GC/g. A notable finding was the presence of RVs, detected in 60% of the samples (36/60) with an average concentration of 5.05 ± 0.4 Log GC/g. HAstVs were present in 25% of the samples (15/60) with a mean concentration of 4.00 ± 1.83 Log GC/g (Table S2). All samples tested negative for HEV and HAV. In addition to the analysis of enteric viruses and viral faecal indicators, mussels were also tested for respiratory viruses. However, all samples were negative for SARS-CoV-2, IAV, and RSV.

Contamination with two or more viruses was reported in a substantial proportion of the analysed mussel samples. Specifically, 52% of the samples (31/60) were contaminated at least with two enteric viruses. The most prevalent viral co-detection occurred between HuNoV GI and RV (29/60 samples). Additionally, 20 samples showed evidence of contamination by three viruses: in most of these cases, the observed co-contamination comprised HuNoV GI, HuNoV GII, and RV. In one sample, the simultaneous presence of HuNoV GI, RV, and HAstV was detected. Finally, the most complex pattern of quadruple viral presence was identified in 12 samples, with simultaneous detection of HuNoV GI, HuNoV GII, RV, and HAstV.

Moreover, an assessment of faecal viral indicators was undertaken. The majority of the samples, specifically 88% (53/60), were positive for somatic coliphages, with an average concentration of 3.62 log plaque forming units (log PFU)/100 g of mussel tissue in positive samples. The levels of somatic coliphages in the analysed mussels revealed a substantial variability, ranging from 2.02 to 5.22 log PFU/100 g. Additionally, crAssphage was detected in 50% of the samples (30/60), with a mean concentration of 3.72 ± 0.6 Log GC/g, indicating significant faecal viral contamination (Table S2).

**Fig. 3:**
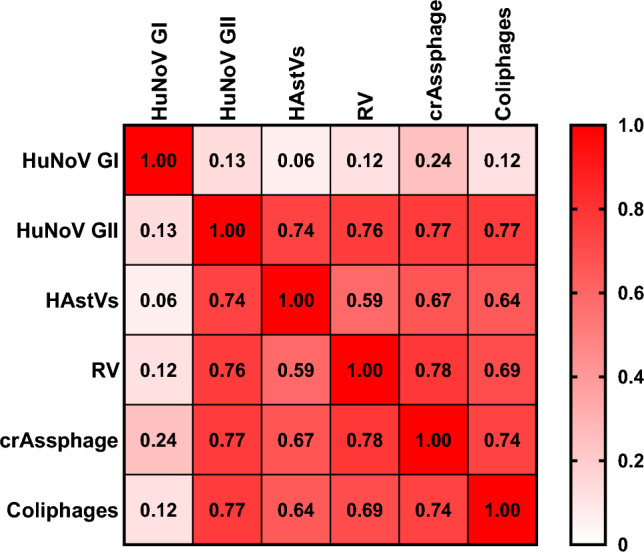
Spearman rank correlation between levels of human enteric viruses and viral faecal contamination indicators in mussels collected in Italy. HuNoV GI, human norovirus genotype I; HuNoV GII, human norovirus GII; HAstV, human astroviruses; RV, rotavirus

Furthermore, Spearman’s rank correlation coefficients (ρ) were calculated between the levels of enteric viruses (*n* = 60, excluding HAV and HEV as they were not detected) and viral faecal indicators (Fig. 3). HuNoV GI showed weak positive correlations with faecal indicators concentrations, with a ρ of 0.24 (*p*-value < 0.05) for crAssphage and a ρ of 0.12 (*p*-value < 0.05) for coliphages.

In contrast, the levels of crAssphage and somatic coliphages showed stronger correlations with the concentration of the other tested enteric viruses. For crAssphage, ρ was 0.77, 0.67, and 0.78 for HuNoV GII, HAstV, and RV concentration, respectively (*p*-values < 0.05). The levels of somatic coliphages yielded similar Spearman’s rank correlation ρ to crAssphage, with 0.77, 0.64, and 0.69 for HuNoV GII, HAstV, and RV concentration, respectively (*p*-values < 0.05).

### Viral Infectivity Approximation by Capsid-Integrity RT-qPCR

**Fig. 4:**
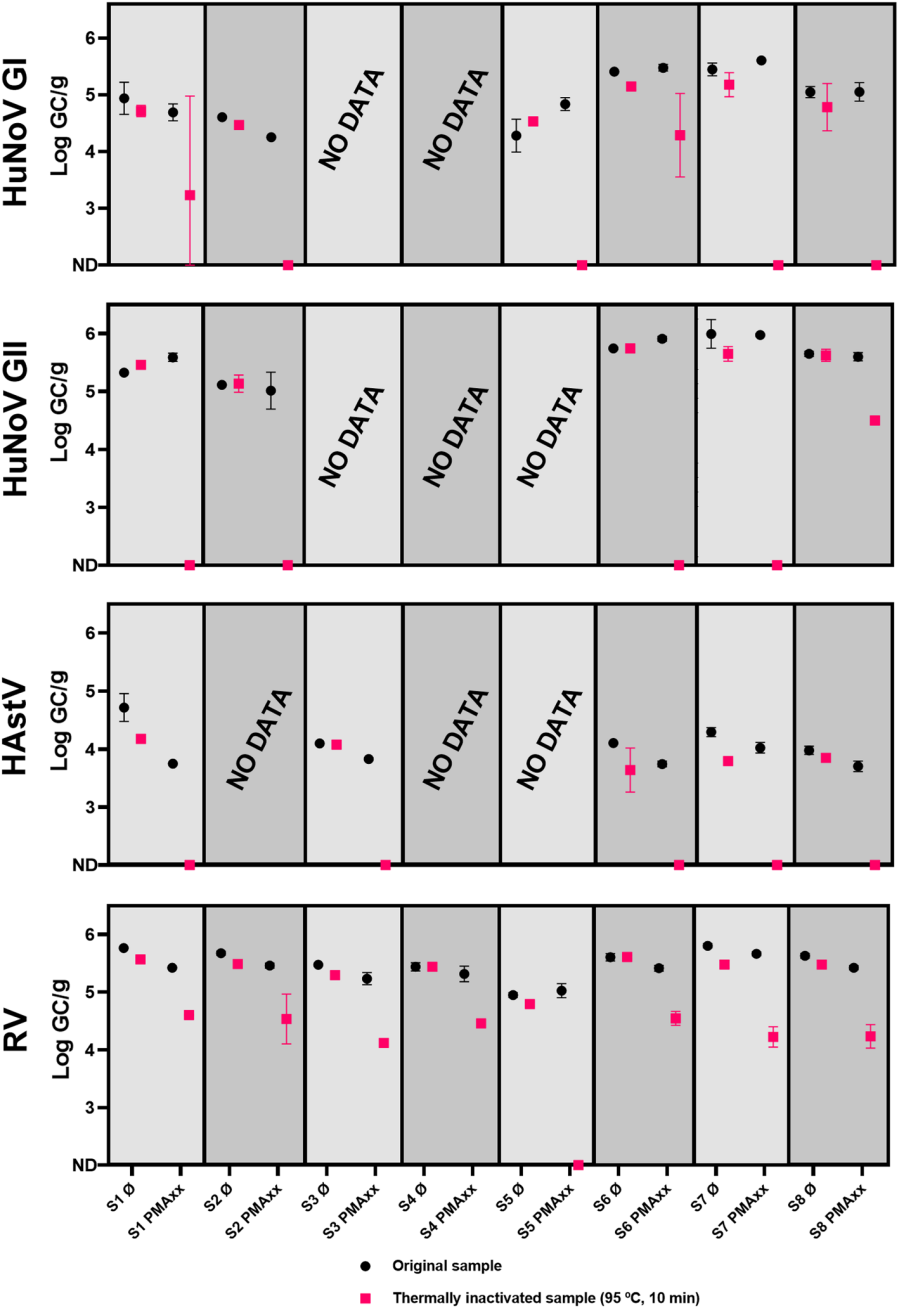
Performance of viability PCR to discriminate between infectious and thermally inactivated viruses in mussels. S1→S8 samples correspond to M21, M27, M28, M29, M38, M41, M42, and M43 samples, respectively. S1→S8 Ø: samples without PMAxx pretreatment; S1→S8 PMAxx: samples with PMAxx pretreatment. HuNoV GI, human norovirus genotype I; HuNoV GII, human norovirus GII; HAstV, human astroviruses; RV, rotavirus

Samples with PCR-confirmed co-presence of HuNoV GI and GII, RV, and HAstV were selected for the capsid-integrity RT-qPCR assay to check for potential viral infectivity. All samples were thermally inactivated in parallel and processed as capsid-compromised control samples. The obtained results followed the same pattern: in all cases, PMAxx pretreatment reduced the PCR signal more in the thermally inactivated subsample than in the original sample (Fig. 4). The original sample generally showed similar results to non-treated samples, thus suggesting the presence of intact-capsid viruses. PMAxx pretreatment completely removed the PCR signal in 6/6, 4/5, and 5/5 of the thermally inactivated subsamples for HuNoV GI, HuNoV GII, and HAstV, respectively. In the case of RV, given the high initial concentration values, the RT-qPCR signal was only completely removed in one out of eight samples. Notably, by replacing “non-detected” raw data concentration results with the LoD/L value of the RT-qPCR specific to each virus, the average reduction in concentration in samples treated with PMAxx compared to untreated samples was 0.13 ± 0.29 Log GC/g. On the other hand, the average reduction in those same heat-inactivated samples was significantly higher (*p*-value < 0.05), at 2.02 ± 0.95 Log GC/g, even when accounting for the underestimation caused by correcting cases where the virus was not detected after PMAxx treatment (Fig. 4).

### Presence of Antibiotic Resistance Genes in Bivalve Molluscan Shellfish

**Fig. 5:**
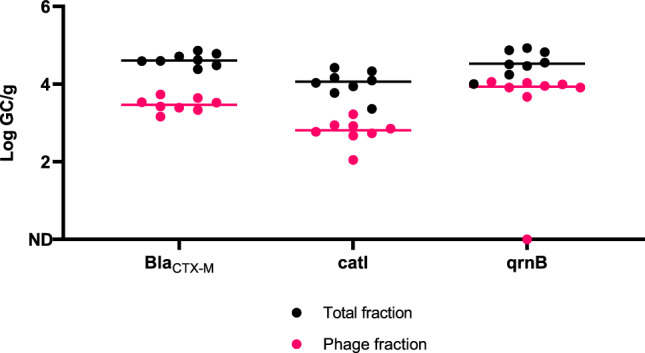
Levels of antibiotic resistance genes (ARGs) in the total and the bacteriophage purified fraction of mussels (*n* = 8) expressed in Log GC/g. Each line represents the median value of each gene

The presence of ARGs in both the total and phage fractions was assessed in samples exhibiting the highest presence of viable somatic coliphages (*n* = 8) (Table S5). The *bla*_CTX-M_ gene was detected at concentrations of 4.63 ± 0.04 Log GC/g in the total fraction and 3.51 ± 0.16 Log GC/g in the phage fraction. Furthermore, the *catl* gene was found at concentrations of 4.08 ± 0.16 Log GC/g and 2.63 ± 0.25 Log GC/g in the total and phage fractions, respectively. Lastly, the *qnrB* gene was identified at concentrations of 4.43 ± 0.14 Log GC/g in the total fraction and 3.92 ± 0.03 Log (GC/g) in the phage fraction (Fig. 5).

## Discussion

Evaluating the incidence of viral pathogens, as well as ARGs in Italian mussel samples, represents a critical endeavour in understanding the safety and contamination risks associated with BMS consumption. The findings of this work reveal a notable prevalence of viral contamination and ARGs in commercially available mussels, despite the limited number of samples analysed for the latter. These results raise concerns about the safety of these commonly consumed seafood products. The obtained results regarding the high incidence of HuNoVs in mussel samples are particularly concerning. HuNoVs have been identified as the causative agents of a significant number of foodborne outbreaks, as emphasized in the latest report from the European Food Safety Authority (EFSA) (EFSA & ECDC, 2023a). Interestingly, we observed a distinct pattern in the prevalence of HuNoV genotypes, with HuNoV GI being significantly more prevalent (77%) than HuNoV GII (40%). This finding contrasts with the research conducted by Fusco et al. (2017) in the Campania region, where a different distribution pattern was observed, with HuNoV GII (39.7%) detected more frequently than HuNoV GI (10.8%) in mussel samples. This difference could be due to using non-retail mussels that had not undergone a depuration step, combined with the greater resistance of HuNoV GI to breakdown during wastewater treatment processes (Suffredini et al., 2011). Also, the 10-year gap between studies introduces variation in the biological, social, environmental, and technological context when conducting the sampling. Besides, while the majority of documented HuNoV outbreaks have been associated with HuNoV GII genotypes, results in this work are in line with other previous research indicating that HuNoV GI strains are more prevalent in bivalve molluscan shellfish (Marsh et al., 2018; Rajko-Nenow et al., 2014; Verhoef et al., 2010).

The detection of RV in a significant proportion of mussel samples (60%) was noteworthy. This prevalence appears to be higher compared to similar research conducted in Italy. For instance, in a study by Fusco et al. (2017), RV was detected in only 16.87% of samples. Notably, the viral load observed in the present study, approximately 10^5^ GC/g, is comparable to the levels reported by Fusco et al (2017). The identification of HAstV in approximately 25% of the samples underlines a consistent presence of this virus in Italian bivalve molluscan shellfish, aligning with previous research findings. In particular, Fusco et al. (2017) reported a prevalence of HAstV at 32.53% in mussels from southern Italy, and in a more recent investigation, this percentage decreased to 20.8% (Fusco et al., 2019). Thus, even if some regional variability exists, these results confirm the stability of RV and HAstV contamination patterns in BMS in Italy over time, as evidenced by consistent detection rates across different studies conducted in recent years.

In this study, HAV was not detected in any of the analysed samples, which contrasts with some earlier reports. For instance, Fusco et al. (2019) reported a low prevalence of HAV-positive BMS, amounting to 8.9% over a three-year study period in Southern Italy. However, there are studies that reported higher HAV prevalence. Pavoni et al. (2022) detected HAV in a substantial proportion of BMS samples (69.23%), while La Rosa et al. (2020) found HAV in 13.7% of BMS samples in the Campania region during 2015–2018. Indeed, the decrease in HAV prevalence observed in this study may reflect a shifting trend in response to the HAV outbreak that occurred during 2017–2018, as documented by the SEIEVA (Servizio Epidemiologia e Sorveglianza Epidemiologica delle Epatiti Virali Acute) (Mele A. et al., 2021). Between January and June 2023, only six HAV cases were reported in Campania, indicating a decreased prevalence compared to previous years. This reduction in clinical cases is consistent with the findings of our study, where no HAV was detected in mussels, aligning with the broader regional epidemiological data (ISS, 2023).

Our study did not detect HEV in any of the analysed mussel samples. This result is consistent with the findings reported by Fusco et al. (2019) and aligns with the limited presence of HEV reported in previous research conducted in Italy (Aprea et al., 2018; Fusco et al., 2017; La Bella et al., 2020; La Rosa et al., 2018; Macaluso et al., 2021). Additionally, no clinical cases of HEV were reported in Campania during the first half of 2023, further supporting the observed results in mussels collected from the region (ISS, 2023).

Additionally, this study revealed a high incidence, 52% of the samples, of BMS contaminated with two or more enteric viruses. Notably, 38% of these samples simultaneously harboured all four targeted viruses. This high prevalence of multiple contamination is consistent with the findings from other studies in Europe. For instance, Pavoni et al., (2022) documented that a high number of BMS samples analysed in a 6-year survey were contaminated by more than one virus. The presence of multiple viruses or virus strains in a single sample can potentially lead to more severe clinical manifestations, the occurrence of dual or mixed infections, and even facilitate the emergence of new recombinant strains (Lees, 2000; Polo et al., 2015).

Due to the microbiological risk associated with the presence of enteric viruses, this study also aimed to assess the levels of somatic coliphages and crAssphage in BMS samples. Coliphages are commonly found in locations with faecal contamination (Grabow, 2001; IAWPRC & Water Microbiology Study Group, 1991), and numerous studies have suggested using coliphages as viral indicators (S. W. et al.,. , 1985; Grabow, 2001; IAWPRC, 1991; Kott, 1966; Lucena et al., 2004; Ueda & Horan, 2000). Besides, in recent years, crAssphage has been proposed as an optimal viral faecal indicator (Bivins et al., 2020; Farkas et al., 2019; García-Aljaro et al., 2017; Kitajima et al., 2014; Symonds et al., 2019; Tandukar et al., 2020; Wu et al., 2020).

In the present study, average levels of 3.62 Log PFU/100 g of mussel ranging from 2.02 to 5.02 log PFU/100 g were reported for somatic coliphages (Table S2), similar to those reported by Olalemi et al. (2016) (4.60 Log PFU/100 g of mussel). Regarding crAssphage, prevalence (50%, *n* = 60) was lower than previously reported by Farkas et al. (2019) (79–83%, *n* = 25) and Gyawali et al., (2021) (70.8%, *n* = 24). Moreover, the mean concentration was 3.72 Log GC/g, in line with those reported in the studies cited above.

In this study, we also aimed to correlate the presence of crAssphage and somatic coliphages in BMS samples with various enteric viruses. Both viral indicators showed strong correlations (*ρ* > 0.65, *p*-value < 0.05) with the levels of HuNoV GII, HAstV, and RV. In contrast, they rendered weak correlations with the levels of HuNoV GI (*ρ* = 0.12, *p*-value < 0.05 for the coliphages and *ρ* = 0.24, p-value < 0.05 for crAssphage). The existing literature on the correlation between crAssphage and enteric viruses in bivalve molluscan shellfish is relatively limited. A study conducted by Farkas et al. (2019) reported no significant correlation between crAssphage and HuNoV, indicating the need for further research to better understand the dynamics of crAssphage as a viral indicator of BMS contamination. Regarding somatic coliphages, the obtained results are similar to those reported by Olalemi et al. (2016) for adenovirus F and differ from Legnani et al. (1998) which found no correlation in bivalve molluscan shellfish samples. Cho et al. (2018) reported weak correlations of somatic coliphages presence in BMS growing area water samples with HuNoV GI and HuNoV GII (*ρ* = 0.33, *p*-value < 0.05 and *ρ* = 0.28, p-value < 0.05, respectively). The obtained results suggest that although crAssphage and somatic coliphages correlate with some of the tested viruses, they failed to indicate the presence of HuNoV GI, which, ultimately, was the most prevalent virus in this study. Thus, further research is needed to expand available data and promote novel approaches to mitigate the spread of viral disease through BMS consumption.

Our study, conducted from April to June 2023, also aimed to further investigate the presence of respiratory viruses in BMS. The sampling period coincided with a decrease in reported COVID-19 cases in the region (Statista, 2023) and the onset of the spring season. It is well established that the epidemiological patterns of respiratory viruses, including IAV and RSV, typically exhibit peak incidence during the winter months in temperate regions (García-Arroyo et al., 2022; Jiménez-Jorge et al., 2016). Given the differences in sampling periods and the relatively low number of reported cases during this study timeframe, the absence of detectable respiratory viruses in mussel samples was consistent with our expectations.

In relation to the potential transmission of viral diseases from BMS products to consumers, a subset of samples was reanalysed to assess the viral capsid integrity. Capsid-integrity PCR approaches have been used in recent years to assess the infectious potential of viruses in various matrices in the absence of routine cell culture methods for most of the viruses present in the environment (Leifels et al., 2021). Furthermore, facilities and equipment required for cell culture may be less accessible to microbiology laboratories. Capsid-integrity assays have been validated for enteric and respiratory viruses in BMS samples (Polo et al., 2021; Quijada et al., 2016; Randazzo et al., 2018a; Sarmento et al., 2020). Although earlier observations showed that proteinase K digestion included in the ISO protocol for processing BMS samples can affect capsid integrity and thus virus infectivity (Langlet et al., 2018), this work demonstrates the presence of intact viral particles in the analysed mussels (Fig. 4). Randazzo et al. (2018a) reported that the PMAxx–Triton pretreatment completely prevented RT-qPCR detection of thermally inactivated HuNoV GII in mussels. This finding aligns with our results, which also demonstrated a substantial reduction in PCR-detectable HuNoV GII in thermally inactivated samples. However, for HuNoV GI, Randazzo et al. (2018a) observed that PMAxx–Triton pretreatment only reduced the RT-qPCR signal by approximately 1 log, indicating a less effective reduction compared to our findings where the signal was completely removed in all thermally inactivated subsamples. Similarly, Sarmento et al. (2020) observed a significant reduction of up to 3 logs of the RT-qPCR signal for HuNoV GI and GII in BMS samples, further demonstrating the effectiveness of PMAxx pretreatment. Moreover, our research is the first to test PMAxx pretreatment for HAstV and RV in mussels, highlighting its potential applicability for different enteric viruses. The obtained results suggest the presence and co-presence of intact-capsid enteric virus particles in the selected samples. Therefore, samples positive for both infectious phages (detected by plaque count) and potentially infectious enteric viruses, (detected by PMAxx-RT-qPCR) were identified in this study. This dual presence may represent a significant infection risk for consumers. However, this risk does not necessarily translate into reported outbreaks in the targeted geographical area, as mussels are typically cooked before consumption. Besides, even if there are Italian regions where mussels are traditionally consumed raw, cases of gastroenteritis may remain underreported, as gastrointestinal illnesses are often considered mild and may not prompt visits to medical professionals or hospitals. Nevertheless, the capsid-integrity assay using PMAxx employed in this study has several limitations that need to be acknowledged. First, the method’s effectiveness in accurately distinguishing between infectious and non-infectious viral particles may be compromised when applied to proteinase-treated samples, as enzymatic digestion can alter capsid integrity, leading to potential misrepresentation of viral infectivity. Additionally, while the PMAxx pretreatment demonstrated reductions in RT-qPCR signal for thermally inactivated viruses, the degree of reduction observed, particularly for HuNoV GI, was less than that expected from cell culture methods. This suggests that PMAxx may underestimate the extent of viral inactivation, thereby introducing uncertainty into the estimation of viable virus concentrations. Furthermore, the assay’s reliability in complex matrices such as mussel hepatopancreas extracts, which contain potential inhibitors, has not been fully validated.

On the other hand, significant differences (*p*-value < 0.05) were observed between reductions in thermally inactivated samples (2.02 ± 0.95 Log GC/g) and non-inactivated samples (0.13 ± 0.29 Log GC/g), suggesting that while the technique might not be fully optimized, it demonstrates an ability to discriminate between heat-inactivated and non-inactivated viruses in BMS samples. The observed differences between inactivated and non-inactivated BMS samples, coupled with the presence of culturable somatic coliphages, raise significant concerns about the potential for these samples to contain infectious viruses. These findings highlight the importance of complementing the capsid-integrity assay with additional methods, such as culture-based techniques, to provide a more robust and comprehensive assessment of viral infectivity in food safety evaluations.

Regarding antimicrobial resistance genes, the presence of ARGs in BMS, WWTPs effluents, and other foodstuffs has been widely reported (EFSA & ECDC, 2023b; Kijewska et al., 2023; Pazda et al., 2019). The spread of ARGs needs to be addressed worldwide (Atlas, 2013; Mckenzie et al., 2014), and marine bacteria can act as ARG reservoirs and facilitating the spread of these genes, which may translate in an increased number of potentially resistant human and animal pathogens (Chen et al., 2020; Ramamurthy et al., 2014). Thus, it is important to understand and mitigate their occurrence in different ecological systems. This study has shown the prevalence of three different ARGs from three of the most widely used antibiotic groups. These groups have been identified to exert selective pressure, contributing the maintenance and propagation of ARGs in the environment (Luo et al., 2010; Mao et al., 2015; Pruden et al., 2006). This work revealed that *bla*_CTX-M_ and *qrnB* levels were present at high concentrations in the analysed mussel samples. In line with this, Brandão et al. (2017) detected the bla_TEM_ gene in 91.3% of the oyster samples (*n* = 23) at levels of 3.81 Log GC/g. Interestingly, these levels closely resemble those obtained in this study for the bla_CTX-M_ gene, which recorded 4.63 ± 0.04 Log GC/g in the total fraction of the hepatopancreas concentrates. Few studies have addressed the detection of ARGs in the phage fraction of mussels. In this study, bla_CTX-M_ gene was detected at 3.51 ± 0.16 Log GC/g in the phage fraction, higher concentration than previously reported (Blanco-Picazo et al., 2023). This work results reflect that there may be ARGs in phage particles enriched from mussel samples that had previously shown high somatic coliphage levels. This finding should not be underestimated considering the ubiquity of phages and their resistance to depuration processes (Jebri et al., 2021), but further studies would be needed to fully confirm ARGs presence in the bacteriophages, as the shellfish matrix may contain inhibitors that could affect DNase efficiency, potentially allowing a portion of free DNA to persist in the phage fraction. Finally, authors acknowledge that results in this work regarding the capsid-integrity approach and ARG detection should be interpreted with caution as preliminary findings due to the limited sample size. Future studies with larger sample sizes would be needed to validate these results and strengthen the conclusions.

## Conclusion

Overall, this study provides a valuable analysis of viral contamination and ARGs presence in mussels from the Campania region of Italy. The high prevalence of human pathogenic enteric viruses, such as HuNoVs, RVs, and HAstVs, along with significant levels of viral faecal contamination indicators like somatic coliphages and crAssphage, highlights considerable public health risks. Besides, the capsid-integrity RT-qPCR assay revealed the potential infectiousness of detected viruses, underscoring the need for optimizing advanced detection methods even though cell culture should remain the gold standard for assessing viral infectivity. Finally, the notable presence of ARGs in both total and phage fractions suggests that phages may facilitate the spread of antibiotic resistance, adding another layer of concern. These findings emphasize the importance of continuous surveillance and strategic interventions to mitigate risks associated with contaminated bivalve molluscan shellfish, ultimately aiming to enhance food safety and protect consumer health.

## Supplementary Information

Below is the link to the electronic supplementary material.

Supplementary file1 (DOCX 48 KB)

## Data Availability

No datasets were generated or analysed during the current study.
