# Supplementary material for: Presence of Potentially Infectious Human Enteric Viruses and Antibiotic Resistance Genes in Mussels from the Campania Region, Italy: Implications for Consumer’s Safety
Source: Food Environ Virol. 2025 May 15;17(2):28. doi: 10.1007/s12560-025-09635-5 (PMC12081582; doi:10.1007/s12560-025-09635-5)
Supplement: Supplementary file 1 — Supplementary file1 (DOCX 48 KB) [file 12560_2025_9635_MOESM1_ESM.docx]

**Supplementary material**

**Presence of potentially infectious human enteric viruses and antibiotic resistance genes in mussels from the Campania Region, Italy: implications for consumer’s safety.**

Iolanda Venuti^1^, Enric Cuevas-Ferrando^2*^, Irene Falcó^2,3^, Inés Girón-Guzmán^2^_,_ Marina Ceruso^1^, Tiziana Pepe^1^, Gloria Sánchez^2^

^1^Department of Veterinary Medicine and Animal Production, University of Naples Federico II, Via F. Delpino, n. 1, 80137 Naples, Italy

^2^Institute of Agrochemistry and Food Technology, IATA-CSIC, Av. Agustín Escardino 7, Paterna, Valencia 46980, Spain.

^3^Department of Microbioogy and Ecology, University of Valencia, Valencia, Spain.

**Table S1.** Primers, probes, PCR conditions, limit of quantification per litre (LOQ/L), and limit of detection per litre (LOD/L) for all targeted viruses in this work.

**Table S2.** Enteric viruses (HuNoV GI, HuNoV GII, HAstV, and RV), and viral faecal indicators (crAssphage and somatic coliphages) levels in mussel samples collected in Italy (n = 60) in Log GC or PFU/g. HuNoV GI, human norovirus genotype I; HuNoV GII, human norovirus GII; HAstV, human astroviruses; RV, rotavirus. ND: not detected. * Represents samples with only one positive RT-qPCR duplicate.

**Table S3**. Primers used for antibiotic resistance analysis.

| **Antibiotic Group** | **Target Gene** | **Oligonucleotide** | **Sequence** | **Ref.** |
| --- | --- | --- | --- | --- |
| β-lactam | Bla _CTX-M_ | FW | TTAGGAARTGTGCCGCTGYA | (Dallenne et al., 2010) |
|  |  | R | CGATATCGTTGGTGGTRCCAT |  |
| Quinolones | qnrB | FW | GATCGTGAAAGCCAGAAAGG | (Kim et al., 2009) |
|  |  | R | ATGAGCAACGATGCCTGGTA |  |
| Chloramphenicol | catl | FW | GGTGATATGGGATAGTGTT | (Yoo et al., 2003) |
|  |  | R | CCATCACATACTGCATGATG |  |

**Table S4.** (RT-)qPCR standard curves for all genomic targets contained in this work.

**Table S5.** Antibiotic resistance genes (ARGs) levels in the total and the phage purified fractions of mussel samples expressed as Log genome copies (GC)/g. ND: not detected.

|  | **TOTAL FRACTION** | | | **PHAGE FRACTION** | | |
| --- | --- | --- | --- | --- | --- | --- |
|  | **ARGs levels (Log GC/g)** | | | | | |
| **Sample** | **bla_CTX-M_** | **catl** | **qnrB** | **bla_CTX-M_** | **catl** | **qnrB** |
| **M22** | 4.59 | 3.94 | 4.47 | 3.16 | 2.67 | 3.91 |
| **M28** | 4.38 | 3.36 | 4.51 | 3.42 | 2.05 | 3.67 |
| **M39** | 4.71 | 4.09 | 4.00 | 3.53 | 2.94 | 3.95 |
| **M41** | 4.48 | 3.77 | 4.55 | 3.52 | 2.92 | 4.03 |
| **M42** | 4.78 | 4.33 | 4.24 | 3.64 | 2.85 | 3.91 |
| **M43** | 4.89 | 4.03 | 4.93 | 3.73 | 3.22 | ND |
| **M47** | 4,62 | 4.16 | 4.87 | 3.33 | 2.77 | 3.99 |
| **M48** | 4.60 | 4.42 | 4.82 | 3.39 | 2.73 | 4.06 |
| **Mean** | 4.63 | 4.01 | 4.55 | 3.46 | 2.77 | 3.93 |

**References**

Stachler, E., Kelty, C., Sivaganesan, M., Li, X., Bibby, K., & Shanks, O. C., 2017. Quantitative CrAssphage PCR Assays for Human Fecal Pollution Measurement, Environ Sci Technol. 51, 9146–9154. <https://doi.org/10.1021/acs.est.7b02703>

Jothikumar, N., Cromeans, T. L., Robertson, B. H., Meng, X. J., Hill, V. R., 2006. A broadly reactive one-step real-time RT-PCR assay for rapid and sensitive detection of hepatitis E virus, J. Virol. Methods, 131, 65–71. 10.1016/j.jviromet.2005.07.004

CDC, 2020. CDC 2019-novel coronavirus (2019-nCoV) real-time RT-PCR diagnostic panel. [Https://Www.Fda.Gov/Media/134922/Download. Accessed October 2020](Https://Www.Fda.Gov/Media/134922/Download.%20Accessed%20October%202020).

World Health Organization, 2009. CDC protocol of real-time RT-PCR for swine influenza (H1N1). World Health Organization, Geneva, Switzerland. http://www.who.int/csr/resources/publications/swineflu/CDCrealtimeRTPCRprotocol_20090428.pdf. Accessed 8 May 2009.

Sanghavi, S. K., Bullotta, A., Husain, S., & Rinaldo, C. R., 2012. Clinical evaluation of multiplex real-time PCR panels for rapid detection of respiratory viral infections, J Med Virol. 84, 162–169. https://doi.org/10.1002/JMV.22186

Dallenne, C., Da Costa, A., Decré, D., Favier, C., Arlet, G., 2010. Development of a set of multiplex PCR assays for the detection of genes encoding important β-lactamases in Enterobacteriaceae. J. Antimicrob. Chemother. 65, 490–495. https://doi.org/10.1093/jac/dkp498

Kim, H.B., Park, C.H., Kim, C.J., Kim, E.-C., Jacoby, G.A., Hooper, D.C., 2009. Prevalence of Plasmid-Mediated Quinolone Resistance Determinants over a 9-Year Period. Antimicrob Agents Chemother. 53, 639–645. https://doi.org/10.1128/aac.01051-08

Yoo, M.H., Huh, M.-D., Kim, E., Lee, H.-H., Jeong, H.D., 2003. Characterization of chloramphenicol acetyltransferase gene by multiplex polymerase chain reaction in multidrug-resistant strains isolated from aquatic environments. Aquac. 217, 11–21. https://doi.org/10.1016/S0044-8486(02)00169-2
